# Supplementary material for: Prevalence and associated factors of postpartum anemia after cesarean delivery in public hospitals of Awi zone, North West Ethiopia, 2023; a cross-sectional study
Source: PLoS One. 2025 Jan 24;20(1):e0311907. doi: 10.1371/journal.pone.0311907 (PMC11760021; doi:10.1371/journal.pone.0311907)
Supplement: Attachment — Submitted filename: PONE-D-24-07591.pdf [file pone.0311907.s004.pdf]

PLOS ONE

Magnitude and Associated Factors of Postpartum Anemia After Caesarean Delivery in Public Hospitals of Awi Zone, North West Ethiopia, 2023; A Cross-Sectional Study.

--Manuscript Draft--

**Manuscript Number:**	PONE-D-24-07591
**Article Type:**	Research Article
**Full Title:**	Magnitude and Associated Factors of Postpartum Anemia After Caesarean Delivery in Public Hospitals of Awi Zone, North West Ethiopia, 2023; A Cross-Sectional Study.
**Short Title:**	Magnitude and Associated Factors of Postpartum Anemia After Caesarean Delivery in Public Hospitals of Awi Zone
**Corresponding Author:**	Dawit Misganaw Belay, MSc<br>Assosa University<br>Assosa, Benishangul Gumuz ETHIOPIA
**Keywords:**	postpartum, anemia, cesarean delivery, Awi zone, Ethiopia
**Abstract:**	Background: Anemia is a serious global public health problem, especially in developing nations. Anemia during pregnancy is appropriately recognized, whereas postpartum anemia especially after cesarean delivery in Ethiopia has received very little attention. Therefore, this study to assess the magnitude and associated factors of postpartum anemia after cesarean delivery in public hospitals of Awi zone, North West Ethiopia, 2023.<br>Method: A hospital-based cross-sectional study was conducted among 395 mothers who gave birth by cesarean delivery from April 1 - 30, 2023. Data were collected using a pretested checklist. A simple random sampling technique was used to select study participants. Then the data were entered into EPI-data version 4.6 and exported to the SPSS version 25 for analysis. A logistic regression model was fitted to assess the association between outcome and explanatory variables. Variables with a p-value of 0.25 or less in bivariable analysis were candidates for multivariable analysis and P-value < 0.05 in multivariable analysis was considered to declare a result as statistically significant in this study.<br>Result: the magnitude of postpartum anemia after cesarean delivery was 18.9% (95% CI (15.1, 23.1)) with a response rate of 97.97%. Being primipara (AOR=0.47,95%CI=0.24,0.92), indication for current C/S (malpresentation) (AOR=0.29,95%CI =0.09,0.90), having pre-operation hemoglobin level <11g/dl (AOR=14.5;95% CI=4.11,51.16) and having medical complication during current pregnancy (AOR=5.95,95%CI=1.88,18.83) were significantly associated with postpartum anemia after cesarean delivery.<br>Conclusion: The findings of the study show that the magnitude of postpartum anemia after cesarean delivery is a mild public health problem. Therefore, promoting the benefits of early detection and management of pregnancy complications such as predelivery anemia and medical complications is crucial.
**Order of Authors:**	Gebretsadek Habtamu, MSc
	Asmare Talie, MSc, Assistant professor
	Tinsae Kassa, MSc, Assistant professor
	Dawit Misganaw Belay, MSc
**Additional Information:**	
**Question**	**Response**
**Financial Disclosure**<br><br>Enter a financial disclosure statement that describes the sources of funding for the work included in this submission. Review the submission guidelines for detailed	The author(s) received no specific funding for this work.

Powered by Editorial Manager® and ProduXion Manager® from Aries Systems Corporation

requirements. View published research articles from *PLOS ONE* for specific examples.<br><br>This statement is required for submission and **will appear in the published article** if the submission is accepted. Please make sure it is accurate.<br><br>**Funded studies**<br>Enter a statement with the following details:<br>• Initials of the authors who received each award<br>• Grant numbers awarded to each author<br>• The full name of each funder<br>• URL of each funder website<br>• Did the sponsors or funders play any role in the study design, data collection and analysis, decision to publish, or preparation of the manuscript?<br><br>Did you receive funding for this work?	
**Competing Interests**<br><br>Use the instructions below to enter a competing interest statement for this submission. On behalf of all authors, disclose any competing interests that could be perceived to bias this work—acknowledging all financial support and any other relevant financial or non-financial competing interests.<br><br>This statement is required for submission and **will appear in the published article** if the submission is accepted. Please make sure it is accurate and that any funding sources listed in your Funding Information later in the submission form are also declared in your Financial Disclosure statement.<br><br>View published research articles from *PLOS ONE* for specific examples.	The authors have declared that no competing interests exist.

**NO authors have competing interests**<br><br>Enter: *The authors have declared that no competing interests exist.*<br><br>**Authors with competing interests**<br><br>Enter competing interest details beginning with this statement:<br><br>*I have read the journal's policy and the authors of this manuscript have the following competing interests: [insert competing interests here]*<br><br>* typeset	
**Ethics Statement**<br><br>Enter an ethics statement for this submission. This statement is required if the study involved:<br><br>• Human participants<br>• Human specimens or tissue<br>• Vertebrate animals or cephalopods<br>• Vertebrate embryos or tissues<br>• Field research<br><br>Write "N/A" if the submission does not require an ethics statement.<br><br>General guidance is provided below. Consult the submission guidelines for detailed instructions. **Make sure that all information entered here is included in the Methods section of the manuscript.**	Ethical approval was obtained from the Debre Markos University, College of Medicine and Health Science, Institutional Research Ethics Review Committee (IRERC) with Reference number HSC/RCS/144/11/15). Also, A permission letter was secured from Amhara Regional Health Bureau and each hospital. Moreover, individual consent was not applicable since it is a retrospective study of medical records (record review). The ethics committee waived the requirement for informed consent. Finally, the confidentiality of the information and privacy of study participants was maintained.

Powered by Editorial Manager® and ProduXion Manager® from Aries Systems Corporation

**Format for specific study types**<br><br>**Human Subject Research (involving human participants and/or tissue)**<br>• Give the name of the institutional review board or ethics committee that approved the study<br>• Include the approval number and/or a statement indicating approval of this research<br>• Indicate the form of consent obtained (written/oral) or the reason that consent was not obtained (e.g. the data were analyzed anonymously)<br><br>**Animal Research (involving vertebrate animals, embryos or tissues)**<br>• Provide the name of the Institutional Animal Care and Use Committee (IACUC) or other relevant ethics board that reviewed the study protocol, and indicate whether they approved this research or granted a formal waiver of ethical approval<br>• Include an approval number if one was obtained<br>• If the study involved *non-human primates*, add *additional details* about animal welfare and steps taken to ameliorate suffering<br>• If anesthesia, euthanasia, or any kind of animal sacrifice is part of the study, include briefly which substances and/or methods were applied<br><br>**Field Research**<br><br>Include the following details if this study involves the collection of plant, animal, or other materials from a natural setting:<br>• Field permit number<br>• Name of the institution or relevant body that granted permission	
**Data Availability**<br><br>Authors are required to make all data underlying the findings described fully available, without restriction, and from the time of publication. PLOS allows rare exceptions to address legal and ethical concerns. See the PLOS Data Policy and FAQ for detailed information.	Yes - all data are fully available without restriction

A Data Availability Statement describing where the data can be found is required at submission. Your answers to this question constitute the Data Availability Statement and **will be published in the article**, if accepted.<br><br>**Important:** Stating 'data available on request from the author' is not sufficient. If your data are only available upon request, select 'No' for the first question and explain your exceptional situation in the text box.<br><br>Do the authors confirm that all data underlying the findings described in their manuscript are fully available without restriction?	
**Describe where the data may be found in full sentences. If you are copying our sample text, replace any instances of XXX with the appropriate details.**<br><br>• If the data are **held or will be held in a public repository**, include URLs, accession numbers or DOIs. If this information will only be available after acceptance, indicate this by ticking the box below. For example: *All XXX files are available from the XXX database (accession number(s) XXX, XXX.).*<br>• If the data are all contained **within the manuscript and/or Supporting Information files**, enter the following: *All relevant data are within the manuscript and its Supporting Information files.*<br>• If neither of these applies but you are able to provide **details of access elsewhere**, with or without limitations, please do so. For example:<br><br>*Data cannot be shared publicly because of [XXX]. Data are available from the XXX Institutional Data Access / Ethics Committee (contact via XXX) for researchers who meet the criteria for access to confidential data.*<br><br>*The data underlying the results presented in the study are available from (include the name of the third party*	All necessary information and datasets used are available from the corresponding author without restriction.

and contact information or URL).<br>• This text is appropriate if the data are owned by a third party and authors do not have permission to share the data.<br><br>* typeset	
Additional data availability information:	Tick here if the URLs/accession numbers/DOIs will be available only after acceptance of the manuscript for publication so that we can ensure their inclusion before publication.

Powered by Editorial Manager® and ProduXion Manager® from Aries Systems Corporation

Manuscript

Click here to access/download;Manuscript;Manuscript on PPA 1.docx

Magnitude and Associated Factors of Postpartum Anemia After Caesarean Delivery in Public Hospitals of Awi Zone, North West Ethiopia, 2023; A Cross-Sectional Study

Gebretsadek Habtamu[1], Asmare Talie[1], Tinsae Kassa[1], Dawit Misganaw Belay[2]*,

[1] Department of Midwifery, College of Health Sciences, Debre Markos University, Debre Markos, Ethiopia.

[2] Department of Midwifery, College of Health Sciences, Assosa University, Assosa, Ethiopia.

* Corresponding Author: Dawit Misganaw Belay: davemisganaw.dm@gmail.com.

ABSTRACT

Background: Anemia is a serious global public health problem, especially in developing nations. Anemia during pregnancy is appropriately recognized, whereas postpartum anemia especially after cesarean delivery in Ethiopia has received very little attention. Therefore, this study to assess the magnitude and associated factors of postpartum anemia after cesarean delivery in public hospitals of Awi zone, North West Ethiopia, 2023.

Method: A hospital-based cross-sectional study was conducted among 395 mothers who gave birth by cesarean delivery from April 1 - 30, 2023. Data were collected using a pretested checklist. A simple random sampling technique was used to select study participants. Then the data were entered into EPI-data version 4.6 and exported to the SPSS version 25 for analysis. A logistic regression model was fitted to assess the association between outcome and explanatory variables. Variables with a p-value of 0.25 or less in bivariable analysis were candidates for multivariable analysis and P-value < 0.05 in multivariable analysis was considered to declare a result as statistically significant in this study.

Result: the magnitude of postpartum anemia after cesarean delivery was 18.9% (95% CI (15.1, 23.1)) with a response rate of 97.97%. Being primipara (AOR=0.47,95%CI=0.24,0.92), indication for current C/S (malpresentation) (AOR=0.29,95%CI =0.09,0.90), having pre-operation hemoglobin level <11g/dl (AOR=14.5;95% CI=4.11,51.16) and having medical complication during current pregnancy (AOR=5.95,95%CI=1.88,18.83) were significantly associated with postpartum anemia after cesarean delivery.

Conclusion: The findings of the study show that the magnitude of postpartum anemia after cesarean delivery is a mild public health problem. Therefore, promoting the benefits of early detection and management of pregnancy complications such as predelivery anemia and medical complications is crucial.

Keywords: postpartum, anemia, cesarean delivery, Awi zone, Ethiopia

1. INTRODUCTION

The World Health Organization (WHO) defines anemia as a condition in which the number of red blood cells or the concentration of hemoglobin within red blood cells, is lower than normal(1). Although there is no universally consensual definition of postpartum anemia (PPA), it can be deduced from the definitions offered by various scholars, depending on the duration of the postpartum period. It can be defined as Hgb < 10 g/dl, Hgb < 11g/dl, and Hgb < 12g/dl cut-off values within the first 48 h of delivery, at 1 week and 6 weeks of postpartum duration, respectively(2, 3).
Postpartum anemia is usually brought on by both chronic iron deficiency that has existed throughout pregnancy and bleeding during childbirth. In the third trimester of pregnancy, due to increased nutrient expenditure for the baby's growth causes iron deficiency (2, 4). On the other hand, the woman’s body mass and her total blood volume the impact of blood loss. It also depends on any further medical issues she might have. For instance, a woman with a cardiac problem experiences greater decompensation with less blood loss (5).

Anemia is a serious global public health problem that particularly affects pregnant and postpartum women (6). Anemia accounts for 7% of maternal mortality due to indirect causes and 2.3% of all causes, with indirect causes accounting for 35% of all causes of maternal deaths globally(6, 7). Even though anemia is a global problem, there are regional variations. Western Sub-Saharan Africa, South Asia, and Central Sub-Saharan Africa regions had the highest burdens (8). More specifically, the proportion of postpartum mothers who have anemia ranges from 10% to 30% in developed countries and from 50% to 80% in developing countries (1, 9). In East African nations, about 36.5% of postpartum women were especially vulnerable to postpartum anaemia (PPA) (10). The prevalence of PPA in Ethiopia also ranges from 11.6% in Addis Ababa to 58.7% in the Somali Region (11).

Women who undergo a cesarean section may be more vulnerable to postpartum anemia, because of they having a higher risk of PPH than women who give birth vaginally (12, 13). Increasing rate and number of cesarean deliveries are linked with maternal risks like peripheral organ damage, bleeding (which led to postpartum anemia), need for intensive care, long surgery time,

hysterectomy and maternal death (14, 15). It is crucial to evaluate the morbidities related to a cesarean delivery because numerous studies have shown that cesarean sections increase the risk of maternal morbidity, mortality, and high healthcare costs compared to vaginal deliveries.

Evidence shows that caesarean section increases postpartum hemorrhage largely through increased risk of uterine atony and minimally through severed vessels while opening the abdominal cavity (16-18). Postpartum anemia is more likely to occur in pregnant women who have anemia during pregnancy especially in the third trimester, excessive intrapartum blood loss, younger women, and who did not take an iron supplement while pregnant (19). Up to half of the women who miss prenatal iron supplements develop anemia within 48-hours after delivery (20).

Additionally, Postpartum anemia (PPA) is associated with poor quality of life, palpitations, an increase in maternal infections, exhaustion, diminished cognitive function and postpartum depression. These outcomes may result in poor mother-child bonding, an inability to care for and breastfeeding infant, or slow baby development (1, 21, 22).

Despite positive progress being made in many countries to reduce maternal mortality, there is still evidence of a persistent increase in the rate of indirect causes. Several studies have been conducted on anemia during pregnancy, however, these studies have provided limited information about magnitude of postpartum anemia especially among women undergoing caesarean section even when the global trends show increasing caesarean section rate. In light of this, little is known regarding the postpartum anemia among postpartum women in Ethiopia particularly among women undergoing caesarean section has not been studied. This study aimed to assess the magnitude and associated factors of postpartum anemia after Caesarean delivery in public hospitals of Awi zone, North West Ethiopia, 2023.

2. METHODS AND MATERIALS

2.1 Study Area and Period

This study was conducted in Awi Zone public hospitals. which is part of Amhara regional state northwest Ethiopia, which has eleven distincts and three-town administrations. There are five hospitals in Awi Zone (Injibara general Hospital, Chagni primary Hospital, Dangila primary Hospital, Gimjabet primary Hospital and Jawe primary Hospital) and the Zone has a total population of above one million as population census conducted by central statistical agency of Ethiopia in 2007 and located in North-western part of Ethiopia and which is far 477 km from Addis Ababa a capital city of Ethiopia(23). The Study was conducted from April 1 to April 30, 2023.

2.2 Study design

Hospital based cross-sectional study design was conducted in public hospitals of Awi zone.

2.3 Population

All mothers who gave birth by caesarean delivery in public Hospitals of Awi Zone were our source population. And all mothers who gave birth by caesarean delivery during the last two years and fulfilling the inclusion criteria in public Hospitals of Awi Zone were our study population.

2.4 Eligibility criteria

All cesarean deliveries during the last two years (January 1, 2021 to December 31, 2022) in public Hospitals of Awi Zone were included. However, who have pre-operative severe anaemia and those who received blood transfusion were excluded.

2.5 Sampling Methods

Sample size calculation

Using single population proportion formula:

A single population proportion formula used to estimate the sample size and using the following assumptions: from previous study done in Debre Berhan, Ethiopia the proportion of postpartum anemia after Caesarean delivery was 18%. confidence interval of 95%($Z\alpha/2=1.96$) and 4% of marginal error ($d=0.04$). So, the sample size become **355.**

Using double population proportion formula:

Using Epi- info version 7.2 software application using the following assumptions: two-sided confidence interval 95%, power 80% ratio 1, and enter the variables with high AOR and the results are shown below on table.

Table 1: Sample size calculation using double population proportion formula for magnitude and associated factors of postpartum anemia after Caesarean delivery in public hospitals of Awi zone, 2023.

Factors/variables	Proportions		Sample size with 10% non-response rate	Study/References
	P1	P2		
Fetal macrosomia	26.9	5.49	110+11=121	(24)
Pre-delivery/CS anemia	25.8	3.56	98+10=108	
Maternal blood loss	61.4	23.57	62+7=69	(25)
Frequency of ANC visits	31.08	12.77	180+18=198	(26)

Key: P1=Proportion among exposed, P2= Proportion among unexposed

So, with the above inputs, the maximum sample size required for this study was 355. Therefore, the final sample size become **395** study participants including non-response rate.

Sampling procedure

There are 5 Public hospitals. All hospitals (Injibara general Hospital, Chagni primary Hospital, Dangila primary Hospital, Gimjabet primary Hospital and Jawe primary Hospital) were selected. The card numbers of all mothers who gave birth through cesarean section at Awi Zone public hospital for the last two years before the study (January 1, 2021 to December 31, 2022) were traced from the hospital's delivery log book registry and were listed down. A two-year report of birth

through cesarean section is collected from each hospital health management information system and sum up to calculate proportion. And then total sample size was proportionally allocated to each public hospital. Then, a computer generating simple random sampling technique was used until the allocated sample for each facility were fulfilled. **(Figure1)**

Figure 1: Schematic presentation of sampling procedures for the selection of study subjects at Awi zone public hospitals, Ethiopia, 2023

2.6 Operational definition of variable

The extent of postpartum anemia after caesarean delivery as a dependent variable, defined by World Health Organization (WHO) criteria as a postpartum Hgb level of less than 11 g/dL, measured closest to the day of hospital discharge (27).

Incomplete Card: mothers' card which was not contain major information about mother's condition (post CS hemoglobin level, pre-CS hemoglobin level).

2.7 Study Variables

Dependent variable:

- Postpartum anemia after Caesarean delivery.

Independent variable:

- Socio- demographic related characteristics:
- Obstetric related characteristics:
- Medical conditions related characteristics:

2.8 Data collection procedure

A structured data collecting checklists was prepared according to the objectives of the study adapted from relevant literatures(24, 28) in English language. By reviewing charts, necessary adjustment was made to fit the local condition. The main contents of the checklists were including: socio-demographic variables, obstetrics related variables and medical conditions related variables. Two supervisors (BSc midwifes) and Five data collectors (diploma midwifes) were recruited.

2.9. Data quality control

To assure the quality of data, data collectors and the supervisors were trained for one day by the principal investigator on the study checklist, consent form and data collection procedure. Furthermore, quality of data was assured by pretesting checklist. A pretest was done on other health facility one week before the main data collection. Then the checklist was modified based on pretest finding. In addition, the completeness, accuracy and consistency of collected data will be checked on daily bases during the data collection time by supervisors and principal investigator. Supervisor and principal investigator were closely following the data collection process.

2.10 Data processing and analysis

After checking the data manually for completeness and consistency, the data were cleaned, coded and entered using Epi-data version 3.1 statistical Software. Then data were exported to SPSS version 25 statistical Software for analysis. Descriptive statistics were computed for variables using frequencies, percentages, mean and standard deviation. Graphical presentation such as bar graph, line graphs and pie charts were used to present the findings of the study. Both bivariable and multivariable logistic regression analysis were employed to determine association between the independent variables and the dependent variable. Bivariable logistic regression were done to identify relationship between one independent variable and outcome variable. Those variables with p-value of less than 0.25 during bivariable were fitted into multivariable logistic regression model to identify variables independently associate with outcome variable. Odd ratio with 95% confidence interval and p value were calculated. Variables having P-value < 0.05 in the multivariable logistic regression analysis were considered as associated factors for health seeking behavior. The final model fitness was checked using the Hosmer-Lemeshow Goodness of Fit test and multicollinearity test were done to check the relationship between independent variables.

2.11. Ethical consideration

Ethical approval was obtained from the Debre Markos University, College of Medicine and Health Science, Institutional Research Ethics Review Committee (IRERC) with Reference number HSC/RCS/144/11/15). Also, A permission letter was secured from Amhara Regional Health Bureau and each hospital. Moreover, individual consent was not applicable since it is a retrospective study of medical records (record review). The ethics committee waived the

requirement for informed consent. Finally, the confidentiality of the information and privacy of study participants was maintained.

3. RESULTS

3.1 Socio -demographic characteristics of respondents

There were 1995 deliveries in 2021 and 2688 deliveries in 2022, were delivered by CS in five public hospitals of Awi zone. Among 395 sample of mother after CS delivery, 387 were participated in this study with a response rate of 97.97%. The mean age of participants was 28.37(SD ± 5.11), ranging from 16 to 41 years.

Table 2:Socio-demographic characteristics of respondents in public hospitals of Awi Zone, Ethiopia, 2023.

**Variables**	**Category**	**Frequency**	**Percentage (%)**
Age	15-24	89	23
	25-34	244	63
	≥35	54	14
Residence	Urban	170	43.9
	Rural	217	56.1

3.2 Obstetrics related characteristics of mother

Among the total participants, mothers who had ANC follow up were 325(84%). Nearly three-fourth (72.9%) of them were multigravida and two hundred thirty-nine (61.8%) of them were multipara. The majority, 356 (92%) of the current pregnancy were singleton.

The most common type of CS was emergency 318 (82.2%) followed by elective type 69 (17.8%). Among CS delivery, 24 (6.2%) women have one previous CS scar. The most common indications of cesarean delivery were fetal distress (Non-Reassuring Fetal Heart Rate (NRFHR)) 118(30.5%), followed by prolonged labor (abnormal labor) 85(22%), preeclampsia-eclampsia 53 (13.7%), malpresentation 50(12.9%), and previous CS scar 43 (11.1%).

Table 3: Obstetrics related characteristics of respondents in public hospitals of Awi zone, Ethiopia, 2023

**Variables**	**Category**	**Frequency**	**Percentage (%)**
ANC follow up	Yes	325	84
	No	62	16
Gravida	Primigravida	105	27.1
	Multigravida	282	72.9
Parity	Primipara	148	38.2
	Multipara	239	61.8
Utilization of IFA	Yes	315	81.4
	No	72	18.6
Type of Pregnancy	Single	356	92
	Multiple	31	8
Number of previous C/S	None	331	85.5
	1	32	8.3
	>/=2	24	6.2
C/S type	Emergency	318	82.2
	Elective	69	17.8
Indication for current C/S	Fetal distress	118	30.5
	Prior scar	43	11.1
	Prolonged labour	85	22.0
	Multiple pregnancy	21	5.4
	Malpresentation	50	12.9
	preeclampsia-eclampsia	53	13.7
	Other*	17	4.4
Type of C/S (based on uterine incision)	LUST C/S	383	99.0
	Classic C/S	4	1.0
Weight of newborn	Normal	360	93.0
	Low birth weight	11	2.8

	Macrosomia	16	4.1
Antepartum hemorrhage in the current px	Yes	43	11.1
	No	344	88.9
Post-partum hemorrhage (PPH)	Yes	28	7.2
	No	359	92.8
Other*= APH, Failed induction,			

3.3 Medical conditions related characteristics

Among 387 mothers, 20 (5.2%) had pre-operation anemia (there hemoglobin levels were <11 gm/dL), whereas 367 (94.8 %) of them had hemoglobin level of 11 gm/dL and above. 67 (17.3%) of mother had medical complication in current pregnancy. Of these, pregnancy induced hypertension (PIH) was the most prevalent medical complication which accounts 51(56%). **(See Figure 2)**

~~*Figure 2: Distribution of medical complication during current pregnancy among postpartum mother in public hospitals of Awi Zone, Ethiopia,2013*~~

3.4 Magnitude of postpartum anemia after cesarean delivery

Among 387 postpartum mothers who gave birth through cesarean section, 18.9% (95% CI (15.1, 23.1)) were anemic (there hemoglobin levels were <11 gm/dL). Out of 387 reviewed chart 344 (88.9%) women have no complication, while 43 (11.1%) have complication like vaginal bleeding 23 (33.3%), severe abdominal pain 18(26.1%), surgical site infection 17 (24.6%).

Table 4: Magnitude and explanatory variables of postpartum anemia after cesarean delivery in public Hospitals of Awi Zone, Ethiopia 2023.

Variable	Categories	Frequency	Percentage (%)
Post operation Hgb	<11 gm/dl	73	18.9
	>/=11 gm/dl	314	81.1
Did transfuse blood	Yes	14	3.6
	No	373	96.4
	Yes	43	11.1

Any Post operation complication	No	344	88.9
If yes which;	Surgical site infection	17	24.6
	Vaginal bleeding	23	33.3
	Severe abdominal pain	18	26.1
	Other*	11	15.9
Other*= severe headache, nausea and vomiting, DVT, UTI			

3.5 Factors associated with postpartum anemia after Cesarean delivery

Binary logistic regression analysis was applied to identify factors associated with postpartum anemia after cesarean delivery. In a bivariable logistic regression analysis nine variables such as ANC follow up, parity, utilization of IFA, C/S Type, weight of newborn, indication for current C/S, APH, pre-operation hemoglobin and medical complication during current pregnancy were variables that have found to have association with PPA after cesarean delivery at p- value of < 0.25. **(See Table 5)**

But only four variables were significantly associated with postpartum anemia after cesarean delivery in multivariable logistic regression such as parity, indication for current C/S, pre-operation hemoglobin and medical complication during current pregnancy **(See Table 5)**

The study revealed that the odds of postpartum anemia after cesarean delivery were 53% lower among mothers who were primipara compared to those who were multipara (AOR = 0.47, 95% CI = 0.24,0.92).

In contrast to those mothers whose indication for current C/S was fetal distress, the odds of postpartum anemia after cesarean delivery were 71% and 86% times lower among mother whose indication for current C/S were malpresentation and preeclampsia-eclampsia, respectively, when compared with those mothers whose indication for current C/S were fetal distress (AOR =0.29, 95% CI = 0.09,0.90) (AOR =0.14, 95% CI = 0.03,0.56).

In addition, this study revealed that postpartum anemia after CD was strongly associated with a predelivery Hb level; the odds of postpartum anemia after CD were 14.5 times higher among women with predelivery anemia (Hb level < 11gm/dL), compared to those who had normal predelivery Hb level (>/= 11gm/dL) (AOR=14.5; 95% CI=4.11, 51.16).

Furthermore, having medical complication during the last pregnancy was also significantly associated with of postpartum anemia after cesarean delivery, in which the odds of postpartum anemia after cesarean delivery were 5.95 times more likely among postpartum mother with clinically confirmed medical complication as compared to their counterparty (AOR=5.95, 95% CI=1.88, 18.83).

Table 5: Bivariate and multivariable logistic regression analysis, factors associated with postpartum anemia after caesarean delivery in public hospitals of Awi zone, Ethiopia, 2023

		PPA after C/S				
**Variables**	**Categories**	**Yes (%)**	**No (%)**	**COR (95% CI)**	**AOR (95% CI)**	**p-value**
ANC	Yes	56(17.2%)	269(82.8%)	0.55(0.29,1.03)	0.99(0.24,4.10)	0.992
	No	17(27.4%)	45(72.6%)	1	1	
Parity	Primipara	23(15.5%)	125(84.5%)	0.70(0.40,1.20)	0.47(0.24,0.92)	**0.027**
	Multipara	50(20.9%)	189(79.1%)	1	1	
Utilization of IFA	Yes	54(17.1%)	261(82.9%)	0.60(0.32,1.05)	0.51(0.13,1.93)	0.320
	No	19(26.4%)	53(73.6%)	1	1	
C/S Type	Emergency	67(21.1%)	251(78.9%)	2.80(1.16,6.75	0.43(0.13, 1.49)	0.185
	Elective	6(8.7%)	63(91.3%)	1	1	
Weight of Newborn	Normal	65(18.1%)	295(81.9%)	1	1	
	LBW	2(18.2%)	9(81.8%)	1.01(0.21,4.78)	1.45(0.25,8.30)	0.676
	Macrosomic	6(37.5%)	10(62.5%)	2.72(0.96,7.76)	2.78(0.85,9.15)	0.092
Indication for current CS	Fetal distress	28(23.7%)	90(76.3%)	1	1	
	Prolonged labor	19(22.4%)	66(77.6%)	0.92(0.48,1.80)	1.17(0.55,2.45)	0.682
	Multiple px	2(9.5%)	19(90.5)	0.34(0.07,1.54)	0.16(0.02, 1.15)	0.068
	Malpresentation	7(14.0%)	43(86.0%)	0.52(0.21,1.29)	0.29(0.09,0.90)	**0.032**
	Preeclampsia-eclampsia	11(20.8%)	42(79.2%)	0.84(0.38,1.85	0.14(0.03,0.56)	**0.007**
	Other	6(35.3%)	11(64.7)	1.75(0.59,5.17)	1.96(0.59, 6.54)	0.271
APH	Yes	12(27.9%)	31(72.1%)	1.80(0.87,3.69	1.26(0.50,3.18)	0.628
	No	61(17.7%)	283(82.3%)	1	1	
Pre-operation Hgb	<11gm/dL	14(70%)	6(30%)	12.18(4.50,32.98)	14.50(4.11,51.16)	**0.000**
	>/=11gm/dL	59(16.1%)	308(83.9%)	1	1	
Medical complication	Yes	19(28.4%)	48(71.6%)	1.95(1.06,3.58)	5.95(1.88,18.83)	**0.002**
	No	54(16.9%)	266(83.1%)	1	1	

4. DISCUSSION

This study attempted to assess the magnitude and associated factors of postpartum anemia after cesarean delivery in public hospitals of Awi Zone. The study revealed that the magnitude of postpartum anemia after cesarean delivery was 18.9% (95% CI (15.1, 23.1)).

This finding was in line with the study done in Nigeria which reported that magnitude of postpartum anemia after cesarean delivery was 20.8%(29), and in Debreberhan (18%)(30) and in Debre Markos (22.87%)(26). However, the magnitude of PPA after cesarean delivery in this study was higher than other studies conducted in California (7.3%)(28), Karimnagar of India (9.2%)(31), and Uganda (6.79%)(24). This discrepancy might be due to study settings difference, as the above studies in Debreberhan and Debre Markos were conducted at tertiary hospitals. Although, in this study lowest threshold used was less than 11g/dl hemoglobin level but studies conducted in California and Uganda used less than 8 g/dl and 7g/dl instead respectively.

On the other hand, this finding was lower than those of studies conducted in Turkey (50.85%)(32), Saudi Arabia (72.34%)(33), Mumbai(76.5%)(34), Kenya (25%)(9) and Harari of Ethiopia (47.4%)(25). This difference might be due to the difference in severely anemic mothers in prior to operation being excluded from this study, use of different cutoff points to define postpartum anemia, and difference in postpartum time of screening. Due to a lack of consensus on the definition of PPA, different scholars use different cutoff points to diagnose PPA. In addition. Geographical difference might be also another factor for the abovementioned variation. Additionally, Ethiopians are eating foods that do have high iron content such as cereals, “teff injera”(ferment teff flour), and fruits(35).

~~Independent factors significantly associated with postpartum anemia after cesarean delivery were parity (primipara), indication for current C/S (malpresentation & preeclampsia eclampsia), pre operation hemoglobin(<11g/dl) and having medical complication during last pregnancy.~~

The study revealed that the odds of postpartum anemia after cesarean delivery were lower among mothers who were primipara compared to those who were multipara. This finding is consistent with a cross-sectional study conducted in south India which showed that parity of two or more was significantly associated with postpartum anemia(36). This finding is also supported by Cross-

sectional study conducted in at Debre Berhan referral hospital of Ethiopia(30). The reason might be due to in case of multiparity the muscular strength of the uterus reduced due to the loss of collagen fibers, results decreased uterine contraction after birth leads to blood loss.

In this study, the odds of postpartum anemia after cesarean delivery were lower among mother whose indication were malpresentation and preeclampsia-eclampsia, respectively, than those mothers whose indication were fetal distress, The reason may be due to in case of malpresentation and preeclampsia-eclampsia health care providers consciously follow laboring mothers take immediate action soon after the diagnosis due to fear of complication this may reduce predelivery blood loss. The other possible explanation might be due to in the case of fetal distress emergency C/S is mostly performed type of C/S which results more blood loss. According to studies, postoperative complications were found higher in emergency caesarean section as compared to elective caesarean section like postpartum anemia (70% vs. 40%) and postpartum hemorrhage (40% vs. 6%)(37).

In addition, this study revealed that postpartum anemia after CD was strongly associated with a predelivery Hb level; the odds of postpartum anemia after CD were 14.5 times higher among women with predelivery Hb level of less than 11gm/dL, compared to those who had normal predelivery Hb level (>/= 11gm/dL). This finding is consistent with studies conducted in California and India (28, 38). It is also supported by studies conducted in Uganda, Nigeria and Harari of Ethiopia (24, 25, 29). The possible explanation for this might be because of the women who had preoperative anemia were less tolerant of any amount of blood loss during cesarean section. The possible explanation for this might be because the iron deficiency present during the antenatal period continues through the postpartum period also.

In this study, having medical complication during the last pregnancy was also significantly associated with of postpartum anemia after cesarean delivery. The odds of postpartum anemia after cesarean delivery were more likely among postpartum mother with clinically confirmed medical complication. This finding is consistent with a cross sectional study done in Nairobi of Kenya(9) which showed that complications during pregnancy was significantly associated with postpartum anemia after cesarean delivery. This finding is also supported by study conducted in Nigeria (29)

which reported that co-morbidities like hypertensive disorders in pregnancy was statistically associated with postpartum anemia after cesarean delivery.

Limitation of the study

As the study relies on retrospective review of data some important variables which can have potential to affect the treatment outcome were not included.

5. CONCLUSION AND RECOMMENDATION

This study has provided important information regarding the postpartum anemia and associated factors after cesarean delivery. This study indicated that the magnitude of PPA after cesarean delivery is a mild public health problem per the WHO cut-off value for the public health significance of anemia. Parity, indication for current C/S, pre-operation hemoglobin level(<11g/dl) and having medical complication during current pregnancy were factors significantly associated with postpartum anemia after cesarean delivery.

Determining patients under high risk (having predelivery anemia and medical complication during pregnancy) is still important to be alert to reduce postpartum anemia after cesarean delivery. Prevention, early detection and treatment of predelivery anemia, could reduce postpartum anemia after cesarean delivery. Also, health care providers need to consciously follow laboring mothers with medical complications. In addition, management of anemia at the antenatal period is the most crucial strategy in combating PPA after CD and this is highly recommended in all levels of health care system through FMOH. Furthermore, Further research better to be conducted that can address the limitations of this study and design strategies to improve completeness by using prospective study design.

Abbreviations and Acronyms

APH: Antepartum Hemorrhage

ANC: Antenatal Care

CD:	Caesarean Deliveries
CS:	Caesarean Section
Hgb:	Hemoglobin
IFA:	Iron and Folic Acid
PPA:	Postpartum Anemia
PPH:	Postpartum Hemorrhage
SPSS:	Statistical Product Service and Solution

Availability of data and materials

All necessary information and datasets used are available from the corresponding author without restriction.

Competing interest

The authors have declared that no competing interests exist.

Acknowledgments

The authors thank the data collectors, data collectors’ supervisors and administrative staff of all hospitals for their cooperation to conduct this research.

REFERENCES

1. Milman N. Postpartum anemia I: definition, prevalence, causes, and consequences. Annals of hematology. 2011;90(11):1247-53.
2. Gea-Caballero V, Juárez-Vela R, Iruzubieta-Barragán F. The definition, screening, and treatment of postpartum anemia: A systematic review of guidelines. Birth (Berkeley, Calif). 2020.
3. Muñoz M, Peña-Rosas J, Robinson S, Milman N, Holzgreve W, Breymann C, et al. Patient blood management in obstetrics: management of anaemia and haematinic deficiencies in

pregnancy and in the post-partum period: NATA consensus statement. Transfusion medicine. 2018;28(1):22-39.

4. Mendoza M. Postpartum Anemia: Causes and Treatments. 2022.

5. . MN. Postpartum anemia I: definition, prevalence, causes, and consequences. . Ann Hematol. 2011;90(11):1247-53.

6. Organization WH. Nutritional anaemias: tools for effective prevention and control. 2017.

7. Adedokun ST, Uthman OA. Women who have not utilized health Service for Delivery in Nigeria: who are they and where do they live? BMC Pregnancy and childbirth. 2019;19(1):1-14.

8. Gardner W, Kassebaum N. Global, regional, and national prevalence of anemia and its causes in 204 countries and territories, 1990–2019. Current Developments in Nutrition. 2020;4(Supplement_2):830-.

9. Rukiya A, Gachuno O, Machoki J. Determinants of post-partum anaemia–a cross sectional study. Journal of Obstetrics and Gynecology of Eastern and Central Africa. 2015;27(1):33-82.

10. Tusa BS, Weldesenbet AB, Bahiru N, Enyew DB. Magnitudes of Anemia and its determinant factors among lactating mother in East Africa countries; using generalized mixed effect model. Frontiers in Nutrition. 2021;8:401.

11. Seifu B, Yilma D. Prevalence and Associated Factors of Anemia among Lactating Women in Ethiopia from 2010 to 2020: A Systematic Review and Meta-Analysis. BioMed Res J. 2020;5(2):327-42.

12. Al- Zirqi I, Vangen S, Forsen L, Stray- Pedersen B. Prevalence and risk factors of severe obstetric haemorrhage. BJOG: An International Journal of Obstetrics & Gynaecology. 2008;115(10):1265-72.

13. Bateman BT, Berman MF, Riley LE, Leffert LR. The epidemiology of postpartum hemorrhage in a large, nationwide sample of deliveries. Anesthesia & Analgesia. 2010;110(5):1368-73.

14. Kaplanoglu M, Bulbul M, Kaplanoglu D, Bakacak SM. Effect of multiple repeat cesarean sections on maternal morbidity: data from southeast Turkey. Medical science monitor: international medical journal of experimental and clinical research. 2015;21:1447.

15. Berghella V. Up‐ to‐ date version 21.2 repeat cesarean delivery2013.

16. Joseph K, Rouleau J, Kramer M, Young D, Liston R, Baskett T, et al. Investigation of an increase in postpartum haemorrhage in Canada. BJOG: An International Journal of Obstetrics & Gynaecology. 2007;114(6):751-9.

17. Carroli G, Cuesta C, Abalos E, Gulmezoglu AM. Epidemiology of postpartum haemorrhage: a systematic review. Best practice & research Clinical obstetrics & gynaecology. 2008;22(6):999-1012.

18. Kramer MS, Berg C, Abenhaim H, Dahhou M, Rouleau J, Mehrabadi A, et al. Incidence, risk factors, and temporal trends in severe postpartum hemorrhage. American journal of obstetrics and gynecology. 2013;209(5):449. e1-. e7.

19. Bodnar LM, Siega-Riz AM, Miller WC, Cogswell ME, McDonald T. Who should be screened for postpartum anemia? An evaluation of current recommendations. American journal of epidemiology. 2002;156(10):903-12.

20. Bergmann RL, Richter R, Bergmann KE, Dudenhausen JW. Prevalence and risk factors for early postpartum anemia. European Journal of Obstetrics & Gynecology and Reproductive Biology. 2010;150(2):126-31.

21. Haas JD, Brownlie IV T. Iron deficiency and reduced work capacity: a critical review of the research to determine a causal relationship. The Journal of nutrition. 2001;131(2):676S-90S.

22. Wassef A, Nguyen QD, St-André M. Anaemia and depletion of iron stores as risk factors for postpartum depression: a literature review. Journal of Psychosomatic Obstetrics & Gynecology. 2019;40(1):19-28.

23. Authority CS. Population and housing census of Ethiopia. Addis Ababa: Central Statistics Authority. 2007.

24. Sivahikyako SA, Owaraganise A, Tibaijuka L, Agaba DC, Kayondo M, Ngonzi J, et al. Prevalence and factors associated with severe anaemia post-caesarean section at a tertiary Hospital in Southwestern Uganda. BMC pregnancy and childbirth. 2021;21(1):1-8.

25. Abebe GT, Kure MA, Yadeta TA, Roba KT, Amante TD. Immediate postpartum anemia and associated factors among women admitted to maternity ward at public hospitals in Harari Regional State, Eastern Ethiopia: A facility-based cross-sectional study. Frontiers in Global Women's Health. 2022:124.

26. Abebaw A, Gudayu TW, Kelkay B. Proportion of immediate postpartum Anaemia and associated factors among postnatal mothers in Northwest Ethiopia: a cross-sectional study. Anemia. 2020;2020.

27. Geneva S, Organization WH. Haemoglobin concentrations for the diagnosis of anaemia and assessment of severity. Vitamin and Mineral Nutrition Information System. Document Reference WHO. NMH/NHD/MNM/11.1. http://www. who. int/entity/vmnis/indicators/haemoglobin; 2011.

28. Butwick AJ, Walsh EM, Kuzniewicz M, Li SX, Escobar GJ. Patterns and predictors of severe postpartum anemia after C esarean section. Transfusion. 2017;57(1):36-44.

29. Akinlusi FM, Rabiu KA, Durojaiye IA, Adewunmi AA, Ottun TA, Oshodi YA. Caesarean delivery-related blood transfusion: correlates in a tertiary hospital in Southwest Nigeria. BMC Pregnancy and childbirth. 2018;18(1):1-9.

30. Asfaw T, Tesema A. Determinant factors, trend and outcomes of cesarean delivery in Debre Berhan referral hospital, North Shewa Zone, Ethiopia. Pediatric Reports. 2020;12(2):8430.

31. Gayathry D, Guthi VR, Bele S, Vivekannada A. A study of maternal morbidity associated with caesarean delivery in tertiary care hospital. Int J Community Med Public Health. 2017;4:1542-7.

32. Dündar B, Çakmak BD. The prevalence and analysis of risk factors for postpartum anemia in women without prepartum anemia. Haydarpaşa Numune Training and Research Hospital Medical Journal. 2019;59(2):165-70.

33. Mattar G, Alsahafi N, Shami B, Abulkhair S, Alhazmi N, Alsaleh R. Incidence of postpartum anemia among postpartum patients in East Jeddah Hospital. International Journal of Pharma and Bio Sciences. 2019;9(2):39-46.

34. Shidhaye PR, Giri PA, Nagaonkar SN, Shidhaye RR. Prevalence of anemia in the postnatal women at a tertiary care teaching hospital in Mumbai. Journal of Medical nutrition and nutraceuticals. 2012;1(1):54.

35. Mihiretu A. Factors associated with anemia among lactating mothers in subsistence farming households from selected districts of Jimma zone, south western Ethiopia: a community based cross-sectional study. Journal of Nutrition and Food Sciences. 2017;7(3).

36. Selvaraj R, Ramakrishnan J, Sahu SK, Kar SS, Laksham KB, Premarajan K, et al. High prevalence of anemia among postnatal mothers in Urban Puducherry: A community-based study. Journal of Family Medicine and Primary Care. 2019;8(8):2703-7.

37. Raees M, Yasmeen S, Jabeen S, Utman N, Karim R. Maternal morbidity associated with emergency versus elective caesarean section. Journal of Postgraduate Medical Institute. 2013;27(1).

38. Rakesh P, Gopichandran V, Jamkhandi D, Manjunath K, George K, Prasad J. Determinants of postpartum anemia among women from a rural population in southern India. International journal of women's health. 2014;6:395.

 Click here to access/download;Figure;Figure 1.docx

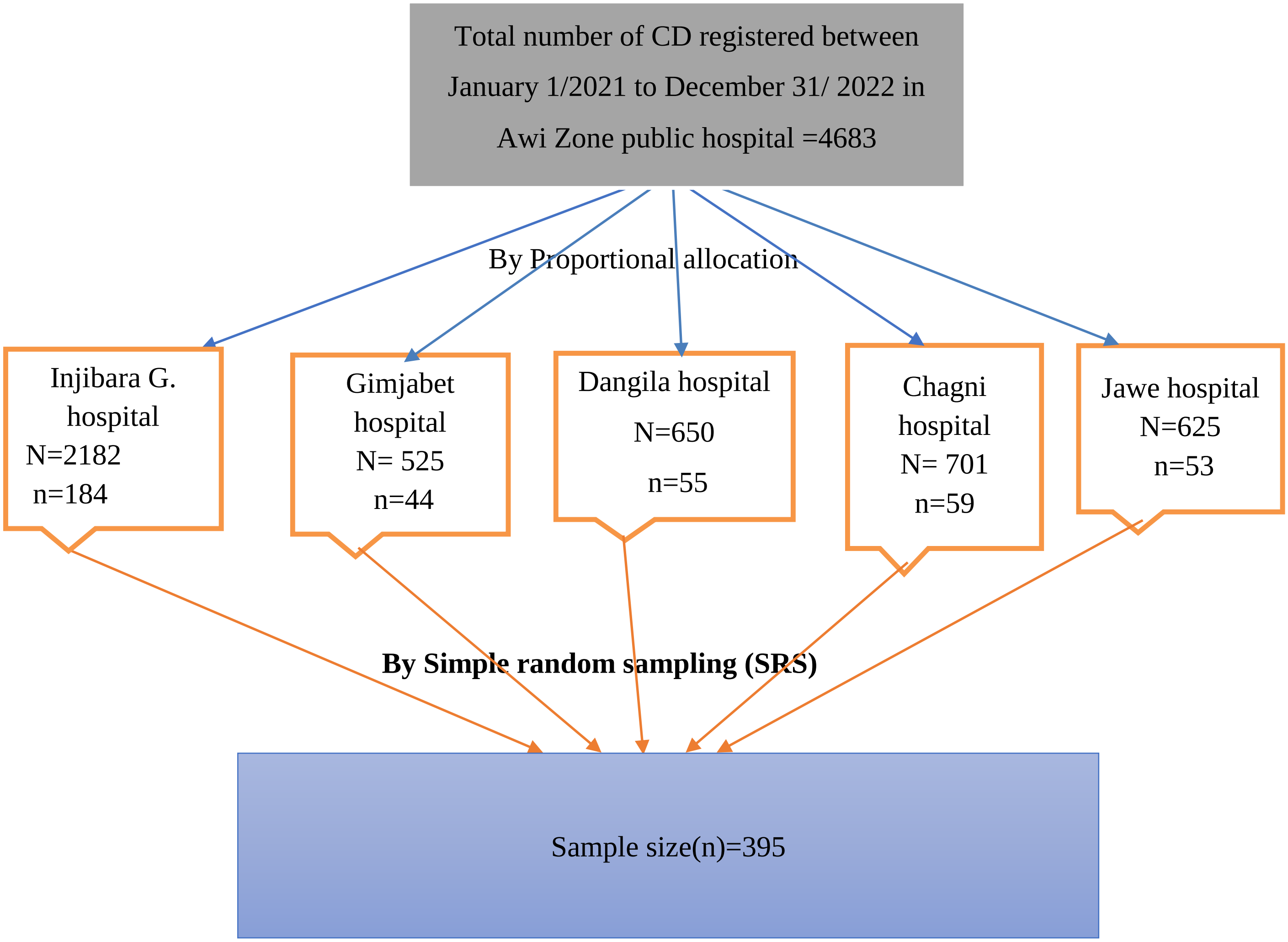

Figure 1:Schematic presentation of sampling procedures for the selection of study subjects at Awi zone public hospitals, Ethiopia, 2023

Figure Click here to access/download;Figure;Figure 2.docx

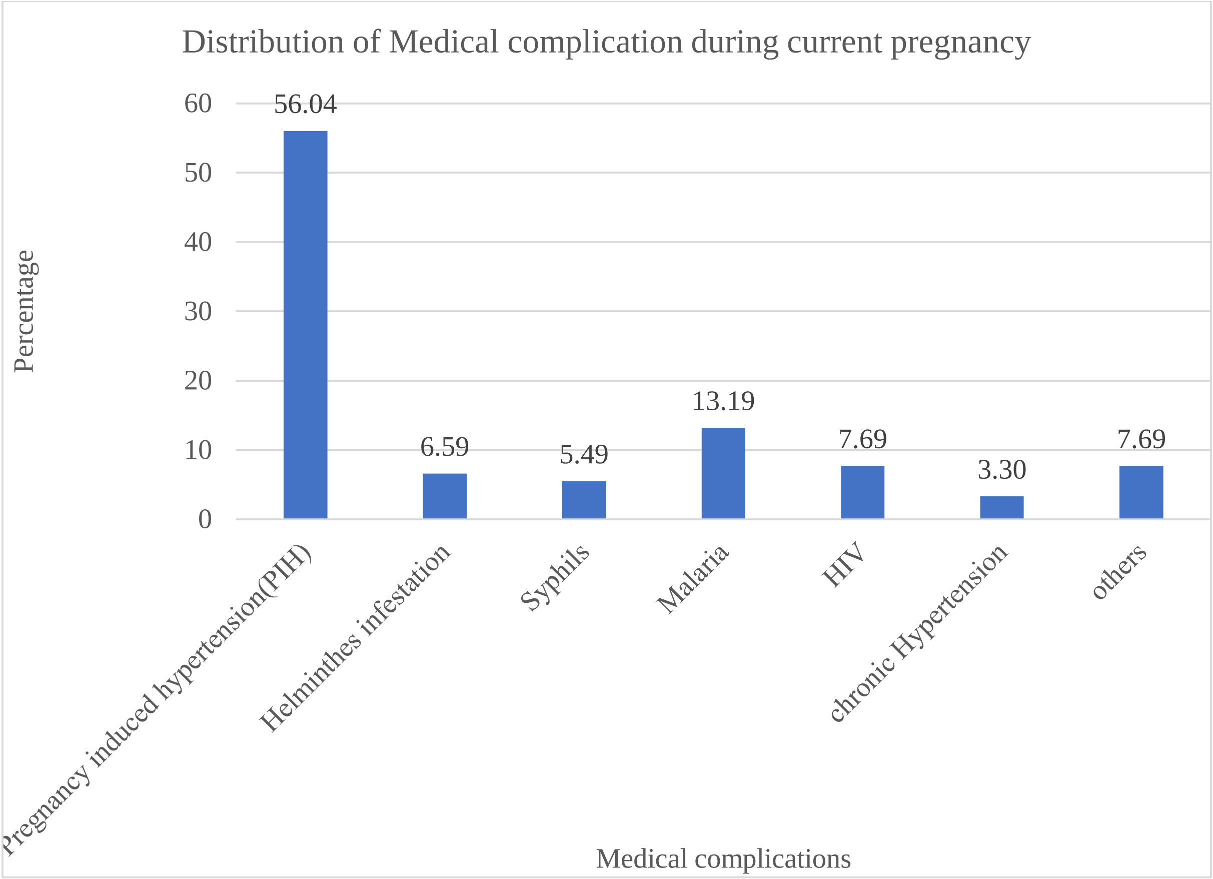

Figure 2: Distribution of medical complications during current pregnancy among postpartum mothers in public hospitals of Awi Zone, Ethiopia,2013
