# Supplementary material for: Prevalence and associated factors of postpartum anemia after cesarean delivery in public hospitals of Awi zone, North West Ethiopia, 2023; a cross-sectional study
Source: PLoS One. 2025 Jan 24;20(1):e0311907. doi: 10.1371/journal.pone.0311907 (PMC11760021; doi:10.1371/journal.pone.0311907)
Supplement: Attachment — Submitted filename: Response to Reviewers.docx [file pone.0311907.s002.docx]

**Date: June 20, 2024**

**Manuscript ID:** PONE-D-24-07591

**“Prevalence and Associated Factors of Postpartum Anemia After Caesarean Delivery in Public Hospitals of Awi Zone, North West Ethiopia, 2023; A Cross-Sectional Study."**

**Dear Editors and reviewers**

Thank you for sending us your valuable comments, which immensely improved our manuscript. We included all the editorial comments raised and enclosed the point-by-point response attached. It is my pleasure to inform you that the manuscript was edited meticulously.

Respectfully,

Dawit Misganaw Belay

Corresponding author

**RESPONSE FOR REVIEWERS**

**RESPONSE FOR REVIEWER #1:**

1. **Comment:** 1. Do you have full confidence to say this study, is cross-sectional?

**Response**: thank you dear reviewer for your valuable concern. Yes, we are sure this is a cross-sectional study. The data is collected from mothers who gave birth by cesarean delivery in public Hospitals of Awi Zone at a **single point in time**. Still, it is a retrospective study of medical records or a review of medical records (secondary data). We believe that to assess the prevalence and associated factors of postpartum anemia after Caesarean delivery the better study design is a cross-sectional study.

- First, we take all cesarean deliveries during the last two years (January 1, 2021, to December 31, 2022) in five public Hospitals of Awi Zone as a study population. (Total Caesarean deliveries registered between January 1/2021 to December 31/ 2022 in Awi Zone public hospital =4683).
- Then, using a simple random sampling technique we have taken 395.
- The data was collected from May 1 to May 30, 2023, by reviewing medical charts/cards of mothers who gave birth by cesarean delivery.

2. **Comment:** What was the basic gap for this study? What if there is no study in the study area and little is known about it? you are well expected to show the impact of little knowledge about PPA after CD?

**Response**: thank you for your scholarly comments and concern. The basic gap for this study is that Several studies have been conducted on anemia during pregnancy, however, these studies have provided limited information about the prevalence of postpartum anemia especially among women undergoing cesarean section even when the global trends show increasing cesarean section rate. Also, little is known regarding postpartum anemia among postpartum women in Ethiopia particularly among women undergoing cesarean section has not been studied. That is why our study aimed to assess the magnitude and associated factors of postpartum anemia after Caesarean delivery. Regarding the impact or significance of the study; this study might provide insight into postpartum anemia after Caesarean delivery to healthcare providers to propose targeted screening and intervention measures. The study will also provide baseline data for concerned governmental entities, policymakers, nongovernmental organizations (NGOs), and other stakeholders to plan and act in the prevention and minimization of postpartum anemia after a cesarean section. Furthermore, the findings of this study might help healthcare providers by providing information on the burden and critical factors that influence postpartum anemia after cesarean delivery. It may also serve as a resource for researchers and educators looking for information on the prevalence and factors associated with postpartum anemia after cesarean delivery.

3. Why do you say this study is cross-sectional? Since, it used the card numbers of all mothers who gave birth through cesarean section at Awi Zone public hospital for the last two years before the study (January 1, 2021 to December 31, 2022) were traced from the hospital’s delivery log book registry and were listed down. A two-year report of birth through cesarean section is collected from each hospital health management information system and sum up to calculate proportion?

**Response**: thank you, dear reviewer, we understand your concern. but it is a cross-sectional study. However, the data were secondary/ retrospective study. The data were secondary data or a review of medical cards of mothers who gave birth by cesarean delivery for the last two years (a retrospective study of medical records/cards). The data were collected from medical records/cards of mothers who gave birth by cesarean delivery **at one point in time (from** May 1 to May 30, 2023).

4. Have you ever tried the reverse of these relations to explore positive association? If, yes why not presented in result section?

**Response**: thank you, in our study both bivariable and multivariable logistic regression analysis were employed to determine the association between the independent variables and the postpartum anemia after cesarean delivery. Bivariable logistic regression was done to identify the relationship between one independent variable and postpartum anemia after cesarean delivery. Those variables with p-value of less than 0.25 during bivariable were fitted into a multivariable logistic regression to identify variables independently/statistically associated with postpartum anemia after cesarean delivery. Finally, variables having P-value < 0.05 in the multivariable logistic regression analysis will be considered as associated factors for PPA. The finding is presented in the manuscript result section (see Table 5). Based on this analysis we got some variables that have **negative and some variables that have positive associations with postpartum anemia** after cesarean delivery.

5. Sections from study design to eligiblity criteria could be deduced in a single paragraph/sentence.

**Response**: thank you for your valuable and constructive comment, comments are accepted and revision was made.

6. Double population proportion should be used for two independent populations and used for study designs compare two different populations. So, why you compute DPPF for this cross-sectional study?

**Response**: thank you for your comment, to get the optimum sample size required for this study we have calculated the sample size using both the single population proportion formula and the double population proportion formula. Then we used the maximum sample size which was calculated from a single population formula. Yes, double population proportion should be used for two independent populations. Using Epi- info version 7.2 statistical software considering two population proportion formula with the assumption using r = 1 (ratio of exposed and unexposed), 80% power, 95% confidence level (Z _α /2_=1.96), where P1 is the proportion of exposed and P2 is the proportion of unexposed. Different variables that are associated with postpartum anemia after cesarean delivery were used.

So, with the above inputs, the maximum sample size we got for this study was 355, which is calculated from the single population proportion formula. Therefore, the final sample size we used was 395 study participants, including the non-response rate.

7. please, be consistent with your study objective/title and/operational definition (for surgical site infection 17 (24.6%)). Or again operationally define this phrase.

**Response**: thank you for your valuable and constructive comment, yes as you see it is not our objective but we have added some other post-cesarean section complications rather than postpartum anemia after CD such as vaginal bleeding, severe abdominal pain, surgical site infection and others to strong our research finding. We accept your comment and we defined surgical site infection operationally. Now, you can see our revised operational definition.

8. Indication for current CS was not significant in bivariate. So, why you use multivariate analysis?

**Response**: thank you for your concern. in a bivariable logistic regression analysis indication for current C/S, was found to have an association with PPA after cesarean delivery at a p-value of < 0.25. you can see the table below rows with bold (indications for current C/S were multiple pregnancies (p-value=**0.162)** and malpresentation(p-value=**0.160).** based on this, those variables with p-values of less than 0.25 during bivariable analysis were fitted/used into a multivariable logistic regression analysis to identify variables significantly associated with PPA after cesarean delivery. That is why we used Indication for current CS in multivariable analysis.

Table; shows Bivariate logistic regression analysis, for Indication for current CS with postpartum anemia after cesarean delivery

| **Variables** | **Category** | **Frequency (%)** | **COR (95% CI)** | **p-value** |
| --- | --- | --- | --- | --- |
| Indication for current C/S | Fetal distress | 118(30.5%) | 1 | 1 |
|  | Prior scar | 43(11.1%) | 0.92(0.48,1.80) | 0.997 |
|  | Prolonged labour | 85(22.0%) | 0.34(0.07,1.54) | 0.819 |
|  | **Multiple pregnancy** | **21(5.4%)** | **0.52(0.21,1.29)** | **0.162** |
|  | **Malpresentation** | **50(12.9%)** | **0.84(0.38,1.85** | **0.160** |
|  | preeclampsia-eclampsia | 53(13.7%) | 1.75(0.59,5.17) | 0.668 |
|  | Other* | 17(4.4%) | 1 | 0.308 |

**Response**: thank you,

9. revise the conclusion section because, a conclusion of a manuscript should be very precise, short and comprehensive.

**Response**: thank you for your valuable and constructive comment, accepted and revision was made.

10. limitation lacks not only treatment outcome, which is not the concern of this study. It lacks variables that can help to generalize the findings.

**Response**: thank you, yes it lacks some important variables that can help to generalize due to the effect of using secondary data. Due to a retrospective review of data, some variables were not available in medical records/cards. That is why we recommend that further research better to be conducted that can address the limitations of this study to improve completeness by using a prospective study design (using primary data). Now, our limitation section has been revised.

11. In general, the manuscript needs critical revision including grammatical correction and keeping the standard based on instruction for authors

**Response**: thank you for your scholarly comments and suggestions. The comments are well noted and revision was made.

**RESPONSE FOR REVIEWER #2:**

1. Title can be simplified as Prevalence of postpartum anemia :An observational study.

**Response**: Thank you for your scholarly comment. The comment is well noted and a revision was made in the title.

2. Abstract contains unknown abbreviation eg AOR?

**Response**: Thank you for your concern. AOR stands for Adjusted Odds Ratio which [is a statistical measure of the association between an exposure and an outcome, after controlling for another variable.](https://www.bing.com/ck/a?!&&p=aeda453bf3656ae9JmltdHM9MTcxODE1MDQwMCZpZ3VpZD0yYzYxYzZmMS0zNGM2LTY2MzctMjFmNi1kNDRhMzU3MjY3MzUmaW5zaWQ9NTc2Ng&ptn=3&ver=2&hsh=3&fclid=2c61c6f1-34c6-6637-21f6-d44a35726735&psq=Adjusted+Odds+Ratio&u=a1aHR0cHM6Ly93d3cuc3RhdG9sb2d5Lm9yZy9hZGp1c3RlZC1vZGRzLXJhdGlvLw&ntb=1) Based on your comment now we have included it under the abbreviations and acronyms section.

3. The major defect in this cross sectional is lacking control group? As it must be compared with non anemic patients so you must compare anemic patients versus non anemic patients as regard age ,parity, previous morbidity and so on

**Response**: Thank you for your valuable and constructive comment. Our study aimed to assess the prevalence and associated factors of postpartum anemia after cesarean delivery in public hospitals of the Awi zone. So, to assess the prevalence of postpartum anemia the better study design we got is cross-sectional study design. That is why we use a cross-sectional study design. Based on this research finding further researchers can investigate or compare anemic as a case and non-anemic as a control **using** **a case-control design as you recommended**.

4. So, this is not cross-sectional study please compare your data to control group otherwise it lacks credibility or significance

**Response**: Thank you for your comment. we understand your concern, but our objective is not only to identify determinants of postpartum anemia after cesarean delivery. Also, even as you said to compare the case with the control first knowing the prevalence of the case is mandatory. That is why we conducted the prevalence and associated factors of postpartum anemia after cesarean delivery. So, in our study comparing cases with control is not our objective.

**RESPONSE FOR REVIEWER #3:**

1. Comment: the conclusion does not refer to or clarify to the paper title i.e. no comment about associated factors.

**Response**: thank you dear reviewer, accepted and revision was made.

2. Sampling procedure:
   Comment: concerning cases who had postpartum hemorrhage or blood transfusion, when was the HB sample collected ? before or after the hemorrhage or before or after transfusion?

**Response**: thank you for your comment, a postpartum Hgb level we used for this study was the Hgb level measured closest to the day of hospital discharge. The variable postpartum hemorrhage is about whether the mother develops postpartum hemorrhage after C/S or not **before Hgb level was measured.** However, the variable blood transfusion was used to show the outcome of postpartum anemia after CS; That means that **after the Hgb level was determined**, whether the mother had transfused blood or not. From the initial, mothers who received blood transfusions before the Hgb level was measured were excluded from the study.

3. Obstetrics related characteristics of mother; The most common type of CS was emergency 318 (82.2%)
   Comment : very high percentage of emergency Cs , what is the reason behind that ? and as mentioned in table 3 : 30 % was due to fetal distress which is also a very high percentage**.**

**Response**: thank you for your comments, there are several reasons why emergency cesarean sections may occur more frequently than elective cesarean sections. This might be due to the following reasons:

- Labor complications: during labor, unexpected complications such as fetal distress, failure to progress, or umbilical cord prolapse may arise.
- Maternal health issues: such as preeclampsia, placenta previa, or placental abruption can develop suddenly during labor, requiring an emergency caesarean section to ensure the safety of both the mother and baby.
- Fetal distress: abnormal heart rate patterns or meconium staining of the amniotic fluid, may prompt healthcare providers to perform an emergency C/S to expedite delivery and prevent potential harm to the baby.
- Failed induction of labor: In cases where attempts to induce labor are unsuccessful or result in complications, an emergency C/S may be necessary to deliver the baby safely.
- Unforeseen circumstances: Sometimes unforeseen events, such as a sudden change in the baby's position or a uterine rupture, can lead to the need for an emergency cesarean section. Overall, emergency cesarean sections are typically performed in response to urgent situations that pose risks to the health and well-being of the mother or baby, while elective cesarean sections are planned for specific medical reasons.

4. Table 3 :
   Comment : Type of pregnancy : should be renamed to number of gestations : singleton or multiple
   Weight of newborn
   Comment : change normal to average
   Comment : change Gravida to Gravidity

**Response**: thank you for your constructive comments. Accepted and revised.

5. Study Area and Period; Comment ; you should mention the number of deliveries annually in the area**.**

**Response**: accepted and revision was made.

6. Table; Comment: why include severe abdominal pain as a complication ? and what was the cause of the pain ?

**Response**: thank you for your valuable and constructive comments, yes, it is not our objective but we include it as an outcome explanatory variable. Severe abdominal pain can be included as a potential complication of a cesarean section due to various reasons. Some possible causes of severe abdominal pain after a cesarean section include: uterine infection (endometritis), adhesions, Internal bleeding, and Surgical complications such as injury to surrounding organs or tissues during the surgery can lead to severe abdominal pain.

7. CONCLUSION AND RECOMMENDATION

   Comment : You should mention that you should reduce the emergency Cs rates as it is related to anemia and elaborate a bit about how you can do that in your setting

**Response**: thank you for your comments, we understand your concern yes as you see emergency C/S rate is high. However, it is not significantly associated with Postpartum anemia in the multivariable logistic regression. That is why we did not consider it in our conclusion and recommendation.

8. English; Comment : the English should be revised before publishing

**Response**: thank you dear reviewer for your valuable and constructive comments

**RESPONSE FOR REVIEWER #4:**

1. Title: Replace the term “Magnitude” with prevalence.

**Response**: thank you dear reviewer, accepted and revision was made.

2. Introduction:
Line 40-42: “It can be defined as Hgb < 10 g/dl, Hgb < 11g/dl, and Hgb < 12g/dl cut off values within the first 48 h of delivery, at 1 week and 6 weeks of postpartum duration, respectively(2, 3). Since you put different three cut-off value, you would cite all the sources. Again write ‘hour’ in full.

**Response**: thank you for your valuable and constructive comments, accepted and revision was made.

3. Line 72: “Additionally, … “ Avoid this conjunction because the earlier paragraph states about attributes of postpartum anemia.

**Response**: thank you for your constructive comments, accepted and avoided from the manuscript.

4. “Despite positive progress being made in many countries to reduce maternal mortality, there is still evidence of a persistent increase in the rate of indirect causes” cite the sources.

**Response**: thank you, dear reviewer, accepted and cited.

5. “Several studies have been conducted 78 on anemia during pregnancy” cite the sources

**Response**: thank you for your valuable and constructive comments, accepted and cited.

6. Materials and Methods:
The authors would gave more detail the study settings (hospitals), including the types of maternal services, estimated number of population being served, number skilled personnel… etc.

**Response**: thank you, accepted and revised. “This study was conducted in Awi Zone public hospitals. There are five hospitals in Awi Zone they are Injibara general Hospital, Chagni Primary Hospital, Dangila Primary Hospital, Gimjabet Primary Hospital and Jawe Primary Hospital)”.

7. How the authors could include all two years cesarean deliveries the settings? Was it census study?

**Response**: thank you for your concern, it is not census but all two years cesarean deliveries are our study population, not our sample. “A two-year report of birth through cesarean section is collected from each hospital health management information system and summed up to calculate proportion. Then, the total sample size was proportionally allocated to each public hospital. Then, a computer generating simple random sampling technique was used until the allocated sample for each facility was fulfilled**.” (See Figure 1)**

8. The authors excluded postpartum women who had pre-operative severe anaemia. But they did not define severe anemia either in introduction or methods sections.

**Response**: thank you, now we have defined it under the method section of our manuscript (see operational definition).

9. Line 123 All the public hospitals in the Zone were included.

**Response**: thank you for your constructive comments, revised.

10. Line 13: “The extent of postpartum anemia after cesarean delivery as a dependent variable, defined by World Health Organization (WHO) criteria as a postpartum Hgb level of less than 11 g/dL, measured closest to the day of hospital discharge (27).” That means you have missed possible causes of anemia that could occur after discharge

**Response**: thank you for your concern, our study is an institution-based cross-sectional not a community-based based because our outcome variable is postpartum Hgb level which is determined through laboratory investigation. However, if it is after discharge it is difficult to know the Hgb level of the mother (determining this Hgb level in an outreach setting or home-to-home is difficult) which is why we conducted a hospital-based study.

11. Line 144: Postpartum anemia after Caesarean delivery what; incidence, prevalence, or what? Please, make it clear and understandable for readers.

**Response**: thank you for your constructive comments, revised. It is the prevalence of postpartum anemia after Caesarean delivery.

12. Each independent variable would be depicted.

**Response**: thank you, dear reviewer. the comment is accepted and now it is revised.

13. Line 176 & 177: “The final model fitness was checked using the Hosmer-Lemeshow Goodness of Fit test 177 and multicollinearity test were done to check the relationship between independent variables.” Revise this sentence.

**Response**: thank you for your constructive comment, now it is revised.

14. Results: Line 188 &189: “There were 1995 deliveries in 2021 and 2688 deliveries in 2022, were delivered by CS in five public hospitals of Awi zone.” a confusing statement.

**Response**: thank you for your valuable comment, we moved to the method section (under study period).

15. The study missed important sociodemographic and dietary or nutritional-related attributes of anemia.

**Response**: thank you dear reviewer for your concern. yes, we missed important sociodemographic and nutrition-related variables due to the retrospective nature of our study. In the case of secondary data, it is difficult to get some important variables in the medical cards of mothers. Due to that after conducting a pretest of the questionnaire we removed those variables not available from the medical card. As you know the main drawback of using secondary data is incompleteness. That is why we put it as a limitation of the study.

16. I suggest he authors to include “Medical conditions related characteristics” in the “Obstetrics related characteristics of mother” because they did not mention any non-medical condition under this section. Two mentioned conditions (anemia during pregnancy and PIH) are obstetric related characteristics.

**Response**: thank you for your valuable comment, we moved it into the obstetrics-related characteristics.

17. The authors would distinguish the degrees of anemia (depending on the severity) in their study that is very important management options and prioritizations.

**Response**:

18. The authors would include the p-values of variables associated in bivariable analysis.

**Response**:

19. The authors did not described “medical related conditions in the last pregnancy” as a variable in the descriptive statistics but they analysed it as independent variable predictor of postpartum anemia.

**Response**: thank you for your concern. However, we have described “medical-related conditions in the last pregnancy” in the descriptive statistics previously under medical complication-related characteristics and now under obstetrics-related characteristics. You can see our revised manuscript.

“Regarding medical complications during last pregnancy, among 387 mothers, 20 (5.2%) had pre-operation anemia (their hemoglobin levels were <11 gm/dL), whereas 367 (94.8 %) of them had hemoglobin levels of 11 gm/dL and above. Sixty-seven (17.3%) of mothers had medical complications in their current pregnancy. Of these, pregnancy-induced hypertension (PIH) was the most prevalent medical complication which accounts 51(56%).”

20. Discussion:You would not compare your finding with the findings from California and Uganda those used Hgb level <8 g/dl and <7g/dl, respectively, a cut-off value.

**Response**: thank you for your valuable and constructive comment, now it is revised.

21. The authors compared and contracted their proportions with incidence rates. For instance reference number 33 states about incidence rate postpartum anemia.

**Response**: thank you for your valuable and constructive comment, now it is removed.

22. The authors would not compare their finding with the proportion of anemia among lactating mothers in subsistence farming households. Different population characteristics and diffirent settings. Reference number 35.

**Response**: thank you for your valuable and constructive comment, However, we used it to justify the difference of our study from other studies, not to compare with it.

23. Line 275-277: Would be included in first paragraph or omitted.

**Response**: thank you for your valuable and constructive comment, now it is removed.

24. General comments: this manuscript has major tense, grammar, punctuation, and sentence errors. So the authors must get help from professional language editors.

**Response**: thank you dear reviewer for your valuable and constructive comments. It is my pleasure to inform you that the manuscript was edited meticulously as much as possible.
