# Supplementary material for: Prevalence and associated factors of postpartum anemia after cesarean delivery in public hospitals of Awi zone, North West Ethiopia, 2023; a cross-sectional study
Source: PLoS One. 2025 Jan 24;20(1):e0311907. doi: 10.1371/journal.pone.0311907 (PMC11760021; doi:10.1371/journal.pone.0311907)
Supplement: Attachment — Submitted filename: Response to Reviewers.docx [file pone.0311907.s005.docx]

**Date: August 5, 2024**

**Manuscript ID:** PONE-D-24-07591

**“Prevalence and Associated Factors of Postpartum Anemia After Caesarean Delivery in Public Hospitals of Awi Zone, North West Ethiopia, 2023; A Cross-Sectional Study."**

**Dear Editors and reviewer**

Thank you for sending us your valuable comments, which have immensely improved our manuscript. We have included all the editorial comments raised and attached a point-by-point response. However, Reviewer 1 did not see our previously revised version of the manuscript and still commented on the first submitted manuscript. It is my pleasure to inform you that the manuscript has been edited meticulously.

Respectfully,

Dawit Misganaw Belay

Corresponding author

**RESPONSE FOR REVIEWERS**

**RESPONSE FOR REVIEWER #1:**

Reviewer #1: MOST OF THE COMMENTS I FORWADED HAVE NOT BEEN COMPLETE. THUS, THE AOUTHORS ARE EXPECTED TO DO SO BEFORE PUBLICATION OF THE MANUSCRIPT.

**Response**: Thank you, dear reviewer, for your valuable feedback. However, most of your current comments were addressed in the revised version of our manuscript, but it seems you are still commenting on the first submitted manuscript, which is why you may still see the same gaps. Please refer to the revised version of our manuscript and the attached response to reviewers. Additionally, some new comments have been incorporated into this revised manuscript.

In addition, comment you raised regarding the figure caption, the manuscript is prepared based on the “PLOS ONE Figure Guideline”; which said “Do not include figures in the main manuscript file. Each figure must be prepared and submitted as an individual file. **Figure captions** must be inserted in the text of the manuscript, immediately following the paragraph in which the figure is first cited (read order). Do not include captions as part of the figure files themselves or submit them in a separate document.”

Thank you once again for your valuable comments and suggestions.

**RESPONSE FOR REVIEWER #3:**

1. thank you for replying to all my comments. I think the paper is now ready to be published
   next time try to use better English and address all points of the title of the paper in the discussion.

**Response**: thank you for your scholarly comments and suggestions.
